# Typhoid Fever, Part 1

**Published:** 1896

**Authors:** Constantine Hering


### TYPHOID FEVER.

A name given to groups of symptoms appearing now and then epidemically, each time in a varied form, so that the yearly fevers assume a character called by this name; the same appears at any time, when exhausted, badly-treated men are heaped up too near each other (ship-fever, camp-fever, war typhus), or when some chronic organic diseases, already almost in an incurable state, assume a similar form as a farewell from this world. In each case different drugs may be indicated, and not one in our store of knowledge ought to be excluded. There is none offering a true and complete resemblance to the pathological form, there are no "specifics" for typhus nor for any of the forms established by the pathologists; only at certain times, in certain places, the same drug may be the main medicine for a while.

The distinction between a so-called "Typhoid" and a "Typhus" is entirely useless in the selection of the medicine. The same is the case with localized and not localized forms. The only advantage is, to be able to distinguish such forms as may be or have oftener been contagious. The prognosis also depends not altogether upon the symptoms, and the dietetic rules may be altered in certain forms. Hence we cannot do without pathology.

Beginners may be somewhat aided by knowing what in certain forms, described under peculiar names, already has been given with success.

**Exanthematic forms**: *Apis. Arnica. Arsen. Bellad. Bryon. Calc. ostr. Carb. veg. Lachesis. Mercur. Mur. ac. Nux. mosch. Phosph. ac. Phosph. Rhus tox. Secale. Stramon.*

**Pectoral forms.** Pneumotyphus: *Ant. tart. Bryon. Carb. veg. Hyosc. Phosphor. Rhus tox.*

**Enteric forms.** Ileotyphus, enteric typhus, typhus abdominalis: *Apis. Arsen. Bellad. Bryon. Calc. ostr. Carb. veg. Cinchona. Colchic. Ginseng. Ipec. Lycop. Mur. ac. Nitr. ac. Nux vom. Phosph. ac. Phosphor. Rhus tox. Secale. Sulphur. Veratr. alb. Phosph. ac.* seems to act chemically. GRAUVOGL, II, 35. *Oxal. ac.* is to be placed next to it, after *Apis*, or followed by it. H. GROSS.

**Bilious form.** Typhus icterodes, typhus biliosus: *Bellad. Cham. Merc.*; after anger: *Chamom.*; with sensitiveness in region of liver: *Bellad.*

**Mucous form.** Typhus pituitosus: *Mercur. Pulsat. Rhus tox.*

**Petechial form.** Hemorrhagic, typhus putridus: *Arnica.* | *Arsenic.* | *Camphor. Carb. veg. Chinin. Chlorum. Mur. ac. Nitr. ac.* | *Sulph. ac.*

| *Arsen.*: when putrid, foul, cadaverously smelling stools; brown, dry, leather-like tongue, extreme prostration.

❙ *Mur. ac.*: fetid stools, intestinal hemorrhage, sopor, so weak that he settles down in bed into a heap.

❙ *Arnica*: foul breath, petechiæ, says there is nothing the matter with him.

Putrid decomposition of all the fluids, tongue parched, dry, brown; bloody, cadaverous, fetid stools: *Rhus tox. Phosphor.*

**Cerebral forms.** Typhus cerebralis, typhus encephalicus: *Arnica. Baptis. Bellad. Bryon. Hyosc. Lachesis. Nux mosch. Opium. Phosphor. Rhus tox. Stramon. Veratr. alb.*

**Versatile forms.** Typhus versatilis: *Bellad. Bryon. Chamom. Cina. Digit. Hyosc. Ignat. Lycop. Mur. ac. Natr. mur. Nux vom. Opium. Phosph. ac. Pulsat. Rhus tox. Stramon. Zincum.*

**Stupid forms.** Typhus stupidus: *Arnica. Arsen. Bellad. Bryon. Carb. veg. Cinchona. Coccul. Helleb. Hyosc. Lachesis. Mur. ac.* ❙ *Nitr. spir. dulc. Nux vom. Opium. Phosphor. Phosph. ac.* ❙ *Rhus tox. Secale. Stramon. Veratr. alb.*; with torpor intermitting: ❙ *Phosph. ac.*; with complete stupor: *Opium.*; depression of nervous system without any other affection, except enlarged spleen: *Coccul.*

**Apoplectic forms.** (Congestive fever.) It is absurd to expect anything from large doses of *Bellad.*; it is murderous to bleed, and foolish to apply ice instead of cloths dipped in cold water, changed every few seconds. If it was not or could not be prevented: ❙ *Glonoine. Gelsemin. Laches. Sanguin.* or *Veratr. vir.* may help if their indications are not overlooked.

Impending paralysis of brain: *Lycop. Opium.* | *Phosph. Zincum.*; impending paralysis of lungs: *Arsen. Carb. veg. Phosphor.*

| *Moschus.*: cannot get the phlegm up, breathing and pulse weaker and weaker, fluids roll audibly down the throat, stools and urine pass unconsciously.

| *Carb. veg.*: paralytic condition, stupor, collapse, dissolution of blood.

---

In the beginning of typhoid fevers, almost any medicine may be given, particularly such as are indicated by the genius epidemicus, or prevailing character of complaints. One third of such patients as come under our treatment from the first, ought to be able to sit up and go out again within a week or two; over another third may last from two to three weeks; hardly one third will run to the full time of six weeks, excepting such cases as have been spoiled by wrong treatment, or have, after feeling unwell, taken *Ricinus oil* or *Citrate of Magnesia.*

---

At the beginning, or in the early stages, the following remedies have been given; with sudden tumultuous symptoms, especially congestive headache: | *Bellad.*; with nosebleed: | *Rhus tox.*; gums bleed: *Mercur.* (in yellow fever: || *Carb. veg.*); lassitude and heavy limbs, with headache, white-covered tongue, loss of appetite, belching: *Bryon.*; furred tongue: *Coccul.*; mouth and tip of tongue dry: *Nux*

*vom.*; gastric symptoms, with acute shooting pain, in different parts of body: *Bryon.*; the same, but with sour, bitter belching and vertigo: *Nux vom.*; in case of looseness: *Bryon. Rhus tox.*; with sour, bitter belching: *Pulsat.*; or with flatulence: *Phosph.*; suspended catamenia and gastric symptoms: *Puls.*; vomiting and copious watery diarrhœa: *Ipecac.*; copious, thin, watery stools, after pain in bowels, with rapid sinking: ❙ *Veratr. alb.*; chilliness on the slightest motion: *Nux vom.*; drowsy, but not able to sleep: ❙ *Bellad.*; erethism: ❙ *Rhus tox.*; stiffness: *Bryon.*; wants to lie down on one spot: *Rhus tox.*; trembling, with weary limbs: *Bellad.*; heat unbearable, but uncovering makes chilly: *Pulsat.*; in the beginning of typhus versatilis: ❙ *Bryon.*; later: *Mur. ac.*; if the so-called nervous symptoms are predominating: *Baptis.* or *Gelsemin.* is better than any of the others.

Not only the duration of the fever ought to be a great deal shorter, but the mortality ought to be considerably less. The latter ought to be restricted to patients with such organic diseases as are developed by the fever, or such relapses as are brought on by neglecting the rules during convalescence.

The stage of **Convalescence** is of the highest importance. Most people, who get well within a week, suppose nothing serious has been the matter with them (compare Mr. Kidd's remarks, BRITISH QUARTERLY, 1847, VI, 97), and do not follow the positive rules, to avoid for the full time of six weeks, all excesses in eating or drinking. The least indulgence may bring on a dangerous relapse. The more appetite they have, the more carefully they ought to limit the quantity as well as the quality. All exertions of mind or body are a great risk; coition very often proves fatal.

During Convalescence, the following have been given with success; in the most complete despair of recovery: *Psorin.*; loss of memory: *Anacard.*; hemicrania: *Ignat.*; rheumatic toothache: *Rhodod.*; appetite will not return: *Psorin. Sulphur.*; ravenous appetite: *Arsen. Pulsat.*; cannot eat, everything tastes bitter: *Pulsat.*; unconquerable bilious vomiting: *Cinchon.*; obstinate vomiting: *Kreosot.*; slow recovery, with diarrhœa: *Cinchon.*; sour diarrhœa: *Rheum.*; from using sour things, with cutting: *Ipecac.*; great sexual desire: *Aloes. Phosph. Psorin.*; tendency to tubercular deposit: *Calc. ostr.*; periostitis of sacrum: *Silic.*; constant, painless, œdematous swelling of lower limbs: *Aur. mur.*, after it failed (right side): *Bellad.*; lower limbs as if paralyzed: *Selen.*; obstinate rheumatism: *Colchic.*; strength alone wanting: *Veratr.*; great prostration: *Psorin.*; marasmus: *Cinchon.*; relapse after overexertion of body: *Rhus tox.*; of mind and body: *Cuprum.*; of mind alone: *Nux vom.*; after a fright: *Ignat.*; angry, passion: *Nux vom.*; continual chilliness and sensitiveness to the slightest draught: *Selen.*; weakening sweats day and night: *Psorin.*; convalescence too slow: *Cinchon.*; unpleasant sensations running downwards: *Guaco.*; upwards: *Fluor. ac. Selen.*; threatens to assume a lingering form: *Arnica.*; feverish feeling, appetite delayed: *Coccul.*; slow protracted cases with mild delirium, restless, anxiety: *Arsen.*

**The sick room** ought to be moderately warm, but aired freely, particularly during the time when the sun shines into it, and always after sunrise. A grapery would be the best place to keep the patient in. Rubbing daily with a towel dipped in cold water, is advisable, and if the patient is used to washings, it may be done all over. Towels are better than sponges.

To disinfect a room or destroy all kinds of bad odor, roast three or four beans of coffee on the stove or a hot plate; all other disinfectants are injurious and useless. Tubs of water under the bed will, in most cases, prevent bed sores. In changing the bed-clothes or shirts, put the clean ones in the sun for one hour, shirts inside out, or rub with the hands what will touch the skin, to take off the peculiar sphere of the laundry. Never interrupt sleep.

Pure cold water is to be allowed as much as the sick person wants, in summer cooled by ice around the outside of the vessel, as melted ice is injurious; stale bread well toasted, but only brown, and while hot put into boiling water, cooled afterwards in an earthen vessel, not in metallic; apples boiled in much water and a few small raisins; some raspberry syrup, not made up artificially; or good rice-water, not seasoned or flavored; no vinegar or lemon may be offered to the sick, unless there is a great desire; barley or oatmeal gruel, according to taste; the so-called Essence of Beef is one of the most injurious things, only does harm, and is not nourishing; beef soup only, is advisable in convalescence, and always with some solid food; never allow young animals or

2

unripe fruit or vegetables. Pure American or Hungarian wine in small doses, but no Port wine. Scraped beef made into a fat lump, salted and fried, or a roasted potato is the best, when beginning to eat. Too much hunger should be moderated by the medicine, and very light and plain food given often, and but little at a time. For complaints after each morsel of food, *Pepsin* may be given; for a total want of appetite, give the indicated medicine, and offer such food as the patient used to eat when a child.

### KEY.

| Alumin.
**|** Alum. p. s.
Amm. carb.
Anacard.
Ant. tart.
**||** Apis.
Arg. nitr.
**||** Arnica.
**||** Arsen.
Astacus.
**||** Baptisia.
**|** Bellad.
Borax.
**||** Bryonia.
**||** Calc. ostr.
**|** Camphor.
**|** Canthar.
Carb. anim.
**|** Carb. veg.
**|** Chamon.
Chin. sulph.
**|**| Cinchon.
|| Coccul.
| Coffea.
**|** Colchic.
Conium.
| Crocus.
Crotal.
| Cuprum.
| Digit.
| Dulcam.
| Ferrum.
Ferr. mur.
Fluor. ac.
Ginseng.
Glonoin.
Guaco.
**|** Helleb.
| Hep. s. c.
**|** Hydroc. ac.
**||** Hyosc.
| Ignat.
Ipecac.
Iris vers.
Jodium.
| Kali carb.
Kreosot.
**||** Laches.
Lachnanth.
Lauroc.
**||** Lycop.
| Mercur.
| Merc. subl.
Moschus.
**||** Mur. ac.
Natr. mur.
Nitrum.

|| Nitr. ac.
|| Nitr. sp. d. *
| Nux mosch.
| Nux vom.
|| Opium.
Oxal. ac.
| Phos. ac.
|| Phosphor.
| Psorin.
|| Pulsat.
|| Rhus tox.
| Secale.
Selen.
| Sepia.
| Silic.
Spongia.
Staphis.
| Stramon.
| Sulph. ac.
|| Sulphur.
| Tarax.
| Veratr.
Zincum.

---

* *Sweet Spirits of Nitre* ought to be prepared in the usual manner, by mixing one part of Nitrous Ether with eight parts of pure absolute Alcohol ; it ought to be kept in small bottles, *and should not redden the cork.*

### CONCOMITANTS AND MODALITIES.

### MIND.

---

### FEELINGS.

I. **Indifference :** || *Arnic.* | *Cinchon.* ;—even to the most loved child : *Phosphor.* ; — to the most loved objects : *Mercur.* ; — to everything : *Secal.* ; — with a kind of insensibility, impelling to rub forehead : *Veratr.* ; — and insensibility, with a pale face : *Cinchon.*

II. **Apathy :** *Veratr. alb.* ; — with indifference : *Cinchon. Phosph. ac.* : — and extreme dullness of senses : | *Nitr. sp. d.*

Does not complain at all : | *Opium* ; no complaint of anything : *Hyosc.* ; unless questioned, he says nothing of his condition, which does not seem dangerous to him : *Colchic.* ; says he feels well, nothing the matter with him ; | *Arnic.* ; thinks he is well : *Arsenic.* ; utters no desire : *Helleb.* ; does not ask for anything, has hardly any of the usual wants : *Nitr. sp. d.* ; no action by will : *Helleb.* Compare, Stupefaction, Stupor.

III. **Anxiety :** *Arsen. Bryon.* ; — with nausea and cold sweat on the forehead while standing : *Veratr.* ; —

with pressure in the heart and tearing pains in the loins, sinking of all the forces but restless, more after than before midnight: || *Rhus tox.*; — and weakness: *Arsen.*; — and frightful dreams: *Arsen.*, on waking from them: *Cinchon.*

I. Does not know what to do with himself, mostly in the third hour of the night: **||** *Arsen.* **|** *Kali carb.*

II. Inexpressible sense of illness in mind and body: *Mercur.*; dejected, debilitated, with aversion to thought: *Bryon.*; either sorrowful, depressed or violently delirious: *Bellad.*; depression of spirits, with timidity: *Secale.*; sadness: *Opium*; hopeless of recovery: *Psorin.*, and certain of death: *Baptis.*; uneasiness with sense of illness: *Opium*; uneasiness of mind and body: *Opium*; fear of being left alone: *Hyosc.* | *Lycop.*; believes he is always alone: *Stramon.*; timid and fearful: *Bellad. Bryon.*; faint hearted, timid, inclined to weep, fears death: *Acon.* | *Arsen. Coccul. Rhus tox. Veratr.*

III. Wants to go from one bed into another: **||** *Arsen. Bellad.* **|** *Calc. ostr.* **|** *Cina.* **|** *Chamom. Hyosc. Mezer.* **|** *Rhus tox. Sepia. Veratr.*

IV. Desire to run away (in later stages): *Bellad.*; and much excited: *Stramon.* Compare Desires.

V. Frightful objects constantly before the imagination while his expression is that of fear and terror; believes he sees dogs, cats, rabbits, approaching from all sides, and that he sees ghosts: *Stramon.*

VI. Fear of death: *Acon. Arsen. Bryon. Coccul. Rhus tox. Veratr.*

### CONATION,

### WITH FEELING OF PAIN.

I. Embarrassed: *Hyosc. Sulphur*; does not know whether he will take what is offered or not, whether to take this or that: *Hyosc.*; suspicious: *Bellad.*; great impatience and despair about pains and bad feelings which he cannot describe: *Ignat.*

II. **Bad humour** : *Rhus tox.*; vexed, irritated, wants to quarrel; *Bryonia*; chagrined: *Nux vom.*; quarrelsome: *Bellad.*; angry disposition: *Bryon. Cinchon.*

III. **Irritable** : *Coccul.*; too sensitive, vexed from questions or gives short answers, easily offended: **|** *Bryon.*; --, nervous with depression of spirits, and intolerance of all impressions on the senses, especially of noises: *Cinchon.*: —. after muttering for a while, can endure neither noise nor contradiction, speaks hastily: *Coccul.*: —, peevish, easily offended: *Bryon.*

### DESIRES.

IV. Desire to get out of bed: *Bryon.*; to change beds: *Arsen. Calcar.*; to escape from bed: *Bellad. Bryon. Hyosc. Stramon.*; to go home: *Bryon.*; to run away: *Bellad. Bryon. Hyosc. Stramon.*; to escape: *Bellad. Opium*; to jump out of bed: *Hyosc.*, restrained and calmed with difficulty: *Zincum*; springs up from bed suddenly: *Bellad. Nux vom.*

V. **Wishes to die,** with indifference to the most loved objects: *Mecur.*; inclined to weep, with fear: *Acon. Bryon. Coccul. Rhus tox. Veratr.*

ACTIONS.

I. **Loquacity,** afterwards stupid and irritable: *Lachnanth.*; talks all the time, or not at all: *Stramon.*; — of the business of the day or the last few weeks: *Bryon.*; — to himself: *Rhus tox.*; constant muttering: *Hyosc. Stramon. Tarax.* Compare Delirium.

II. **Murmuring:** *Laches.*; —, which cannot be understood: *Hyosc. Lycop.*; slow —: *Phosph. ac.*; —, with picking clothes: *Hyosc.*; — loquacity: *Hyosc. Laches.*; with an insensible apathy, hardness of hearing, and a pleasant, happy expression, which looks strange: *Apis.*

III. **Talks** incoherently without any seeming connection of ideas: *Laches. Rhus tox.*; — indistinctly: *Hyosc.*; — nonsense with eyes open: *Hyosc.*; — of dogs, wolves, cattle, soldiers, battles: *Bellad.*; — like a drunken man: *Lycop.*

IV. **Sings** and laughs loud in his delirium: *Bellad.*; — laughs, whistles, recites verses, sings opera pieces: *Stramon.*; whines and don't know why: *Hyos.*; moans loudly: *Mur. ac.*; cries out, suddenly: *Hyosc. Lycop. Stram.*; from time to time: *Arsen.*

V. **Staring** constantly at surrounding objects, with apparent entire self-forgetfulness: *Hyosc.*

VI. Picking the bed clothes: *Arnic. Arsen. Colchic. Hyosc. Lycop. Opium. Psorin. Stramon. Zincum*; with muttering: *Hyosc.*; in delirium: *Rhus tox.*; catching, grasping at flocks: *Lycop. Ph. ac. Zincum.*; playing with his own hands (not picking with them): *Hyosc.*; motion with hands as if they would get things: *Ph. ac.*; groping about with hands: *Opium*; waving hands as if getting things out of the air: *Stramon.*; reaching after objects in the air: *Psorin. Sulphur.*

I. Does foolish things, behaves like a madman: *Hyosc.*; acute mania: *Bellad. Stramon.*; beats and scratches others; the milder others talk to them, the worse they get: *Hyosc.*; abuses those about him: *Hyosc.*; strikes, bites or spits at his attendants: *Bellad.*; strikes his attendants, with fearful outcries; great disposition to bite and tear everything with his teeth, even his own limbs: *Stramon.*; raving with pain in the head: *Arsen.*; never awkward but wondrously dextrous: *Stramon.*

II. **Answers,** hastily: *Bryon. Hepar. Rhus tox.*; — rightly, but in a quick, violent manner, as if angry: *Rhus tox.*; — with indignation: *Pulsat.*; hasty speech: *Bryon. Coccul. Hepar*; if he talks at all, it is quick and hasty: *Arsen.*

AVERSIONS.

III. **Silence**: *Veratr.*; as if averse to everything: *Nux vom.*; sits absorbed in silence: *Opium*; with an unconquerable inclination to sleep: *Coccul.*; obstinate silence, will answer nothing: *Cinchon.*; taciturnity: *China.*; don't want to talk: || *Phosph. ac.*; averse to speaking: *Rhus tox.*; with confusion of head: *Mercur.*; reluctant to answer questions: *Phosph. ac. Rhus tox.*; to speak: *Bellad.*; answers short: *Bryon.*; with "yes" or "no": *Phosph.*; short and incorrectly: *Phosph. ac.*; declines to answer questions: *Arnica.*; answers no questions: *Arsen. Hyosc.*

IV. Averse to speaking: thought is difficult: *Rhus tox.*; scarcely answers, in spite of what is done to in-

(I.) duce him to do so, appears to hear without understanding what is said, or without allowing it to make any impression on him: *Nitr. sp. d.*; permits no one to speak to him: *Veratr.*

II. **Refuses** things offered: *Bellad.*, and remains lying indolent, without sleeping or speaking: *Nitr. sp. d.*

III. **Indolence**; sits in silence: *Coccul.*; — of mind and body; through the day averse to employment or movement; in the evening averse to work, pleasure, speech or movement; extremely uncomfortable; knows not what the matter is with him: *Sulphur.*

Quiet and then suddenly restless: *Chlorum.*; avoids moving about, is dull in his senses, stupid and embarrassed: *Sulphur.*; aversions to all efforts of mind and body: *Cinchon.*

COGNITIONS. Reproduction.

IV. **Unconsciousness**: *Arnica. Arsenic. Bellad. Colchic. Hyosc. Laches. Lycop. Mur. ac. Opium. Phosphor. Rhus tox. Stramon. Zinc.*; — and insensibility, muscles relaxed: *Opium*; — with loss of the function of senses: *Hyosc.*; —, like a deep sleep: *Mercur.*; — with imbecility: *Stramon.*; he knows neither where he is nor what he does: *Pulsat.*; lies with both arms stretched along the sides of body; —; sudden starts: *Canthar.*; perceptions entirely lost, picking at the bed-clothes: *Colchic.*

V. **Insensibility**: *Arsen. Cinchon.*;—with indifference: *Veratr.*; — with stupor, feels neither pain nor pleasure: *Opium*; — and loss of consciousness: *Helleb. Rhus tox.*; — with loss of speech, pulseless-

(I.) ness and cadaverous aspect, while the natural heat of the body is retained, and he is in a sleep-like state, from which he emerges with consciousness and speech : *Mercur.* ; stupidity and embarrassment, avoids moving about : *Sulphur.*

II. **Stupor** : *Apis. Laches. Phosph. ac. Rhus tox.* ; — with murmuring delirium : *Apis.* ; — with profuse sweat : *Kali carb.* ; sinks into a state of apathy or stupefaction, remains perfectly unconscious when spoken to or called, cannot be shaken or roused from his lethargy : *Hyosc.* ; apathy, unconsciousness and stupor, with murmuring delirium : *Apis.* ; takes no notice of what occurs, neither sees, hears nor recognizes his relatives, insensible to external impressions : *Stramon.* (Compare Senses.) — with brown parched tongue as hard as a board, teeth and gums covered with a brown mucus, and the usual mucus dried up to hard crusts : *Rhus tox.* ; feeling of drunkenness, with desire to lie down, or with rush of blood to the head : *Bryon.* ; is conscious of no want except thirst : *Hyosc.* ; silly stupidity, sadness and weakened memory : *Opium* ; loss of thought for the moment ; insensibility, so that he knows not where he is : *Mercur.* ; sensibility entirely benumbed : *Opium* ; insensibility ; is compelled to rub the forehead : *Veratr.* ; giddiness, vanishing of thoughts : *Nux mosch.* ; loss of speech : *Hyosc.* ; utter insensibility : *Psorin. Sulphur* ; cannot be brought to himself : *Nitr. sp. d.* ; when spoken to, (III.) answers properly, but unconsciousness and delirium immediately return : *Arnic. Bellad. Hyosc.* ; stupor soon returns : *Hyosc.*

**Loss of memory** : **||** *Anacard. Hyosc.* ; memory

(I.) weakened: *Opium*; forgets the word while speaking: *Arnic.*; forgets time and place: *Mercur.*; and what he has said: *Mur. ac.*; cannot remember the most recent occurrences: *Rhus tox.*; remembers events only as dreams; almost entire loss of mind: *Veratr.*; dullness, like a want of memory: *Pulsat.*

**Recollection**; mind occupied with things past and present: *Mur. ac.*; does not know those about him, relatives or friends: *Hyosc.*; does not comprehend what occurs, knows neither relatives nor the most familiar objects: *Opium*; now he knows his friends, and again he does not: *Bellad.*

II. Slowness of comprehension when asked a question: *Sulphur*; cannot find the right expression for his ideas, does not remember what has passed: *Coccul.*; thinks rightly but uses wrong words for the correct ideas he intends to express: *Arnic. Graph. Lycop.*; great difficulty in speaking to use the right expressions: *Pulsat.*; talks slowly, answers do not correspond: *Carb. veg.*; either does not understand the questions, or does understand and cannot speak: *Hyosc.*; makes irrelevant answers: *Hyosc. Phosph. ac.*; does not understand questions, does not answer: *Secal.*; when spoken to, is as if awakened from a dream, appears silly, and can only comprehend and answer after a great effort: *Sulphur.*; recognizes what is said to him, but as is after a dream: *Opium*; sits absorbed in silence: *Opium*; sits as if in thought, yet thinks of nothing; like a waking dream: *Arnic.*

III. **Absent - mindedness**; with staggering: *Arsen.*; cannot fix his attention on present objects, or manage his affairs: *Sulph.*; as if absorbed in

(I.) thoughts, and yet a want of ideas : *Rhus tox.*; with insensibility as if intoxicated : *Nux mosch.*; absence of ideas : *Sulphur*; entire self-forgetfulness : *Hyosc.*

PRODUCTION.

II. **Intellect** ; difficult thinking and speech : *Secal.*; — clouded, though he gives correct answers; unless questioned, he says nothing of his condition, it does not seem dangerous to him : *Colchic.*; internal dullness as if sleepy or drunk : *Opium*; obtuse mental operations, with great inclination to sleep : *Mercur.*; mind sluggish, with inability to think : *Carb. veg.*

Slow movement of ideas : *Phosphor. Rhus tox.*, and also of the power of comprehension : *Cinchon.*; answers correctly but slowly : *Nux mosch. Rhus tox.*, and
(III.) reluctantly : *Phosph. ac.*; mental operations slow and difficult : *Rhus tox.*; ideas move slowly and constantly around one subject : *Petrol.*; with confusion of head as if bound : *Carb veg.*; dwells long on his answer before giving it, often does not answer at all : *Nux mosch.*; averse to thinking, debility of mind, vanishing of thought like fainting : *Byron.*; difficulty of thinking, great forgetfulness and dullness in head : *Byron.*; cannot bring two ideas into connection, weak in his intellect : *Sulphur*; inability to think; thoughts
(IV.) cannot be directed or controlled : *Hyosc.*; slow comprehension of ideas : *Opium*; confusion of ideas : *Baptis.*; stupefied condition; sits as if in thought, like a waking dream : *Arnic.*; dullness, stupidity : *Opium. Rhus tox.*, and sopor : *Carb. veg.*; lethargy and stupidity : *Nitrum*; lethargy of the sensorium, a kind of half paralysis of the mental organ : *Nitr. sp. d.*;

(I.) imbecility: *Opium. Stramon.*; idiotic state: *Helleb.*; stupid and disconcerted for many days: *Phosphor.*; with dullness of intellect and all the senses: *Opium*; does not think, with confused heaviness in forehead: *Arnic.*; stupidity with dilated pupils: *Secal.*; cannot comprehend an idea, with headache, painful on waking in the morning: *Phosphor.*

II. **Fixed ideas**; when he has once grasped a thought it cleaves to him, and will not vanish: *Pulsat. Petrol.*; some one idea haunting him, monomania: *Stramon.*; the same disagreeable idea arouses him as soon as he falls into a light slumber: *Calc. ostr.*; great crowd of changing ideas: *Laches. Pulsat.*

III. **Illusions**; as if his body were cut in two, in the middle, as if all surrounding objects were very small, while he himself is very large: *Platin.*; very long and tall: *Pallad.*; believes he sees a large company of people about him, and grasps at them: *Stramon.*; delirious phantasies, in slumber and waking, as if she was on a distant island, had great occupation, was a lady of rank, etc.: *Phosphor.*; speaks to the absent as if they were present, and calls inanimate objects by the names of persons, while he takes no notice of his attendants: *Stramon.*; does not believe to be in his own house: *Opium*; thinks he is in the wrong place: *Hyosc.*; wants to go home: *Byron.*; (IV.) believes he is always alone: *Stramon.*; thinks he is dead; muttering stupor: *Apis. Laches.*; says there is nothing the matter with him: *Arnic.*; thinks he is well: *Arsen.*; illusions of the senses, and imagination: *Hyosc.*; has visions of beauty or terror: *Bellad.*; sees people standing at the foot of the bed: *Bryon.*

I. **Terrible visions**; sees animals which he fears: *Bellad.*; — with fear and desire to hide or run away; *Pulsat.*; hides under bed-covers: *Stramon*; frightful visions: *Carb. veg.*; the 14th day: *Calc. ostr. Stramon*; frightful phantasies, in the evening in bed, with frightened starts on closing the eyes to sleep: *Cinchon.*; sees things all the time: *Bellad. Calc. ostr.*; vivid hallucinations: *Mur. ac.*, of sight, hearing and smell: *Stramon*; with frequent changes of vision, (in T. versat.): *Hyosc.*; absent persons talk to him: *Stramon.*

When closing the eyes, sees persons and events before him that are neither fearful nor anxious, mostly strange faces: *Arsen. Calc. ostr. Carb. veg. Sambuc.*

DELIRIUM.

II. **Mild**: *Arsen. Opium. Phosphor.*; changing with loud talking: *Bellad. Hyosc. Stramon.*; talks about business matters: *Byron.*, with coldness: *Veratr.*, with attempts to run away: *Byron.*; —, in the beginning; only in sleep or on awaking: *Bryon.*; — in early stages: *Bellad.*; second stage: *Hyosc.*; after two weeks: *Calc. ostr.*, —, and makes no complaint, in second stage, with general heat: *Hyosc.*; continued when awake; sees persons who are not, and have not been present: *Hyosc.*

III. **Constant**: *Baptis.*, with congestion to head and face: *Opium*; allowing no rest or sleep: *Mur. ac.*

Low: *Rhus tox.*; murmuring: *Arnic. Hyosc. Lycop. Rhus tox. Stramon.*; slow murmuring: *Phosph. ac.*; muttering: *Bellad. Opium. Veratr.*

After *Calc. ostr.*, if the patient is harassed by mut-

(I.) tering delirium, a tearing and stinging headache, lies in a state of quiet sopor, sometimes interrupted by screaming and scolding; with distended abdomen: *Lycop.* JAHR.

II. **Soliloquizes** much: *Rhus tox.*; constant talking, thinks he is roaming over fields, swimming, lying in the water for hours: *Rhus tox.*; — of old occurrences, with open eyes, and recognizes what is said to him, only as if after a dream: *Opium*; —, with talking of religious things, of fulfilling vows, prayers: *Veratr.*; indistinct loquacity: *Apis. Hyosc.*; loquacity, mild or terrified: *Stramon.*; — about his avocations: *Bryon.*; — very loquaciously, with brilliant eyes and circumscribed redness of the cheeks, afterwards stupid and irritable: *Lachnanth.*; — loquaciously or violently and loudly: **|** *Bellad.*; with singing, laughing and whistling: constant involuntary odd motions; objects appear oblique: *Stramon.*; talks of (III.) ghosts, devils and spirits, which he says surround his bed and afflict him: *Opium.* (Compare Actions.)

IV. **Imagines** to be under control of strangers, and desires to go home: *Bryon.*; goes out of bed, does not feel as if he were at home: *Opium*; impression that he is elsewhere than at home: *Veratr.*; talks of going home: *Bellad. Bryon.*; talks of starting on a journey, wants to be dressed, is ready to go: *Opium*; attempts to get out of bed: *Bellad. Hyosc. Opium. Stramon. Zincum*; attempts to escape: *Bellad. Hyosc. Opium*; inclination to run away, compare Desires.

V. **With visions**: *Stramon.*; changing images from the past or present, keep him active and irritated:

(I.) *Mur. ac.*; frightful objects: *Stramon.* (Compare Frightful visions, under Painful Feelings.)

II. **Furious**: *Bellad. Calchic. Pulsat. Zincum*; at the height of the disease: *Stramon.*; —, amounting to roaring madness: *Zincum*; furibund, with loud talking, laughing and attempts to escape: *Opium*; —, raving, restless, obstinate, objects to sleep, redness and prominence of eyes with intolerance of light: *Bellad.*; — violent, with staring eyes; strikes, bites or spits at his attendants: *Bellad.*; —, cries, strikes at all around: *Canthar.*; cries even to hoarseness and complete loss of voice: *Stramon.*; — with desire to escape from bed: *Bellad. Hyosc. Stramon.*; maniacal, gets up, tries to run away, screams, roars with sunken features, cold feet, quick pulse: *Zincum*; — violent, followed by vomiting and deep sleep: *Secal.*; violent, constant talkativeness, with subsultus tendinum and other movements: *Valeria*; — during the hot stage: | *Arsen.* || *Bellad. Hyosc. Opium. Rhus tox.*; with pain in limbs: *Rhus tox.*

III. **Delirium** with stupidity: *Nux mosch.*; with stupor: *Baptis.*; — with loss of consciousness: *Pulsat.*, after still greater vertigo, like intoxication, with sense of lassitude and weakness: *Secal.*; quiet, with great stupefaction and dullness of head: *Phosph. ac.*; anxiety, headache, noise before the ears, great restlessness, loss of speech, trembling, and anxious sweating: *Arsen.*; violent pain in forehead: *Bellad.*; heat of head: *Camphor*, with open eyes: *Bellad. Hyosc. Stramon.*; on closing eyes, all sorts of frightful phantoms: *Calc. ostr.*; — with sunken features: *Zincum*; — with vomiting, and after it, deep sleep: *Secal.*; —wild,

(I.) alternating with stupor and stertorious breathing, with open mouth and depression of the lower jaw: *Laches. Opium.*; — with hoarseness: *Stramon.*; — with floccilegium: *Phosphor.*; — with subsultus: *Valer.* (See Actions.) — on going to sleep: *Cinchon. Ginseng*; — as soon as he falls to sleep: *Gelsem. Spong.*; with sleeplessness: **||** *Bellad.*; — with deep sleep afterwards: *Secal.*; — with sopor: *Lycop.*; — with cold feet: *Zincum*; — with cold, clammy skin: *Camphor.*

II. **Times of the day**; in the evening, with hasty speech: *Bryon.*; especially at night, about business or previous affairs: *Bryon.*; after midnight: *Kali carb.*; in the morning upon business, with disposition to run away: *Bryon.*

---

III. **Agitation,** restlessness, jumping out of bed, attempts to run away: *Hyosc.*; drunkenness with staggering or indolence; frantic drunkenness: *Nux mosch.*

IV. **Prostration**; with depression of spirits; weakness, with inability to do any work; marked sinking of strength, with inability to move; want of tone in the solids of the body: **||** *Opium*; — of the mind, he cannot bring two thoughts together, as if quite stupid: **|** *Rhus tox.*, and depression of spirits: *Mercur.* **|** *Nuphar.*; with torpor: *Arnic.*; mentally restless, but too lifeless to move: **|** *Baptis.*

V. **Fright**; is frightened easily: *Ignat.*, with nervousness: *Kali carb.*; terrified; *Stramon.*

3

### MENTAL STATES AS CONDITIONS.

Mental exertions cause bodily symptoms; on endeavoring to fix the attention on an object, throbbing in the vortex: *Nux vom.*; great anxiety and worriment of mind: *Calc. ostr.* | *Cupr.*; long grief: *Ignat.* | *Opium*; violent passions: *Chamon.*

### INDEX TO MIND SYMPTOMS.

A.

Absence of ideas, 27, iii
Absent-mindedness, 27, iii
Absent persons, talking with, 30, i
Absorbed in silence, 24, iii
— in thought, 28, iv
Abuses others, 24, i
Actions, 23
Afflicted by ghosts, devils, etc., 31, iii
Agitation, 33
Alone, believes to be, 29, iv
Angry disposition, 22, ii
Animals, approaching him, 21, v
— fear of, 30, i
Answers, but soon is unconscious again, 26, iii
— with difficulty, 27, ii
— slowly, 27, ii
— hastily, 24, ii
— incorrectly, 24, iii
— irrelevantly, 27, ii
— not at all, 24, iii
— reluctantly, 24, iii
Answers in a quick, violent manner, 24, ii
— scarcely, if induced, 24, iv
— short, 22, iii ; 24, iii
Anxiety, 20, iii
— with delirium, 32, iii
Apathy, 20, ii
Asked, comprehends slowly, 27, ii
Attention, cannot be fixed, 27, iii
— endeavoring to fix it, 34
Aversions, 24
— to all efforts, 25, iii
— to employment, 25, iii
— to everything, 24, iii
— to pleasure, 25, iii
— to speaking, 25, iii
— to speech, 25, iii
— to thinking, 25, iii
— to thought, 21, iii
— to work, 25, iii
Avocations, about him, talking, 31, ii
Avoids movement, 25, iii ; 26, i

B.

Bad humor, 22, ii
Beats others, 24, i
Bed, attempts to get out of, 31, iv
— wants to change, 21, iii
— wants to get out of, 21, iii ; 31, iv
— gets out of, 22, iv ; 31, iv
— jumps out of, 33, iii
— surrounded by ghosts, etc., 31, iii
Bites, 24, i ; 32, ii
Business of the day, talks about, 23, i
— in delirium, 31, ii

C.

Calmed with difficulty, 22, iv
Catching flocks, 23, vi
Chagrined, 22, ii
Child, the most loved, indifferent to, 20, i
Cleaving to him, thought, 29, ii
Clouded intellect, 28, ii
Cognitions, 25, iv
Company, 29, iii
Complains of nothing, 20, ii ; 30, ii
Comprehension, 27, ii
Comprehend, cannot, 27, ii ; 29, i
— slowly, 28, iii
Conations with feelings of pain, 22
Confusion of head, 24, iii ; 28, iii ; 29, i
Confusion of ideas, 28, iv
Connection of ideas, without any, 23, iii
Consciousness, loss of ; see Unconsciousness.
Contradiction, cannot endure, 22, iii
Control of strangers, to be under, 31, iv
Cries out, 23, iv
— to hoarseness, 32, ii

D.

Dangerous, his condition does not seem to him, 28, ii
Dead, he thinks he is, 29, iv
Death seems certain, 21, ii
Debility of mind, 28, iii
Declines to answer, 24, iii
Dejected, 21, ii
Delirium, 26, ii ; 29, iii ; 30, ii
— returns after answers, 26, iii
Depression of spirits, 21, ii ; 22, iii ; 33, iv
Desires, 22, iv
— to hide or run, 30, i
— to go home, 31, iv
— to lie down, 26, ii
— to run away, 21, iv
— uttering no, 20, ii
Despair, 22, i
Devils, talks of, 31, ii
Dextrous, 24, i
Die, he wishes to, 22, v
Difficult thinking, 28, ii ; 28, iii

Disconcerted, 29, i
Dreams, as after,
27 ; 28, iv ; 31, ii
Dressed, wants to be, 31, iv
Drunkenness, 26, ii ; 33, iii
Drunk, as if, 23, iii ; 28, i
Dullness,
25, iii ; 27 ; 28, iii ; 28, iv
— of senses, 20, ii ; 25, iii

E.

Embarrassed, 22, i ; 25, iii
Escape, attempts to, 31, iv
— from bed, 22, iv
Excited, 21, iv
Expression, happy, but
strange, 23, ii
— of fear and terror, 21, v

F.

Faces, strange, 30, 1
Fainthearted, 21, ii
Fear, of animals he sees, 30, i
— of being left alone, 21, ii
— and wish to die, 22, v
— of death, 21, ii
— in expression, 21, v
— hides himself,
21, iv ; 30, i
Fearful, 21, ii
Feelings, 20
— he cannot describe, 22, i
Feels well, 20, ii
Fixed ideas, 29, ii
Floccilegium with delirium, 33, i
Foolish things, doing, 24, i
Forgets the word, time,
and place, 27, i
Forgetfulness, 27, i
Fright ; frightened
easily, 33, v
Frightful fantasies, 30, i
— objects, 21, v ; 31, v
— phantoms, closing
eyes, 32, iii
— visions, 30, i
Furibund ; furious delirium, 32, ii

G.

Ghosts, he sees, 21, v
— talks of, 31, iii
Grasping at flocks, 23, vi
— a thought, it cleaves
to him, 29, ii
— at people, 29, iii
Grief, 34
Groping with hands, 26, vi

H.

Hallucinations, 30, i
Harassed by muttering
delirium, 30, iii
Hasty answer, 24, ii
— speech, 24, ii
Haunted by one idea, 29, ii
Hiding himself, 30, i
Himself, cannot be
brought to, 26, ii
Home, as if he were
elsewhere, 31, iv
— talking of going, 31, iv
— wants to go,
22, iv ; 29, iv
Hopeless of recovery, 21, ii
House, not in his own, 29, iii

### I.

Ideas, absence of, 27, iii
— changing, 29, ii
— cannot connect the, 23, iii ; 28, iii
— confused, 28, iv
— crowding ; — fixed, 29, ii
— one haunts him, 29, ii
— slow, 28, ii
— want of, 28, i
Idiotic, 29, i
Illness in mind, 21, ii
Illusions, 29, iii
Images, changing, 31, v
Imaginations, 29, iv ; 31, iv
Imbecility, 25, iv ; 29, i
Impatience, 24, ii
Inability to do any work, 33, iv
— to think, 28, ii ; 28, iii
Indifference, 20, i ; 25, iii
— with wish to die, 22, v
Indignation, answers with, 24, ii
Indolence, 25, iii ; 33, iii
Insensibility, 20, i ; 25, iv ; 26, ii
Intellect, 28, ii
— weak, 28, iv
Intolerance of impression on senses, 22, iii
Involuntary, odd motions, 31, ii
Irrelevant answers, 27, ii
Irritable, 22, iii
Irritated, 24, i
— from seeing images, 31, v

### J.

Journey, talks of starting on a, 31, iv
Jump out of bed, 22, iv

### K.

Knows nothing, 25, iv
— not what the matter is, 25, iii
— not where he is, 25, iv
— not what to do with himself, 21, i

### L.

Lady of rank, she thinks she is, 29, iii
Large, he seems to be, 29, iii
Laughs, 23, iv ; 31, ii
Lethargy, 28, iv
Loquacity, 23, i
Loss of memory, 26, iii
— of mind, 27, i
— of senses, 25, iv
— of speech, 25, v
Loud laughing, moaning, 23, iv
— talking in delirium, 30, ii ; 31, ii

### M.

Mad man, behaving like one, 24, i
Madness, 32, ii
Mania, acute, 24, i
Maniacal delirium, 32, ii
Mental exertions cause bodily symptoms, 34
Memory, loss of, weakened, 26, ii ; 26, iii

Mental operation, obtuse, 28, ii
— operation, slow and difficult, 28, iii
Mildness makes him worse, 24, i
Mind, loss of, 27, i
— occupied with past and present, 27, i
— sluggish, 28, ii
Moans loudly, 23, iv
Monomania, 29, ii
Motion with hands, getting things, 23, vi
Murmuring, 23, ii ; 26, ii ; 30, iii
Muttering, 22, iii; 23, i; 30, iii
— with picking, 23, ii
— when sleeping, 22, iii

N.

Nervous, 22, iii ; 33, v
Noises, intolerable, 22, iii
Nothing the matter with him, 20, ii ; 29, iv
Notice, not taking of attendants, 29, iii

O.

Objects, inanimate, 29, iii
— small, 29, iii
Obstinate, 32, ii
Obtuse mental operations, 28, ii
Odd motions, 31, ii
Offended easily, 22, iii
Old occurrences, 31, ii
One subject, ideas, 28, iii
Outcries, fearful, 24, i

P.

Passions, violent, 34
Past and present, occupies mind, 27, i
Peevish, 22, iii
People, he sees at foot of bed, 29, iv
Permits no one to speak to him, 25, i
Perception, lost, 25, iv
Persons, sees, who are not present, 30, ii
Phantasies, 29, iii
Picking bed clothes, 23, ii, iv ; 25, iv
Place, wrong, believes to be in, 29, iii
Playing with his hands, 23, vi
Prayers, 31, ii
Production, 28
Prostration of mind, 33, iv

Q.

Quarrel, wants to, 22, ii
Questions, slow comprehending, 27, ii
— not understood, 27, ii
— vex him, 22, iii
Quiet, 25, iii
— delirium, 32, iii

R.

Raving, with pain in head, 24, i
— delirium, 32, ii
Ready to go, 31, iv
Reaching after things in air, 24

Recites verses, 23, iv
Recognizes, as after dream, 27, ii ; 31, ii
— nobody, 26, ii
— no friends nor familiar objects, 26, ii ; 27, i
Recollection, 27, i
Refusing things, 25, ii
Reluctant to answer, to speak, 24, iii
Religious things, talking of, 31, ii
Remembers not the most recent, 27, i
— only as a dream, 27, i
Reproduction, 25
Restless, 25, iii
— delirium, 32, ii ; 32, iii
— mentally, 32, iii
Restrained with difficulty from leaving the bed, 22, iv
Roaming, 31, ii
Roaring madness, 32, ii
Roars, 32, ii
Run away, to, 22, iv ; 30, i
— attempts to, 30, ii ; 31, iv
— desire to, 21, iv
— inclination to, 31, iv
— tries to, 32, ii

S.

Sadness, 21, ii ; 26, ii
Says nothing about his condition, 20, ii ; 28, ii
Scolding, 31, i
Scratches others, 24, i
Screaming, 32, ii
Sees things, 30, i
— persons and events, 30, i
Self forgetfulness, 23, v ; 28, 1
Sensibility, benumbed, 26, ii
Sensitive, too, 22, iii
Short and incorrect answers, 24, iii
Silence, 24, iii
— sitting in, 25, iii
— absorbed in, 27, ii
Silly, 27, ii
Singing, 31, ii
— in delirium, 23, iv
— opera pieces, 23, iv
Sits absorbed, 24, iii
— as if in thought, 27, ii, iii ; 28, iv
Slow answers, 28, iii
— comprehension, 27, ii ; 28, ii
— movement of ideas, 28, ii
— mental operations, 28, iii
— talking, 27, ii
Sluggish mind, 28, ii
Soliloquizes, 31, ii
Sopor, 28, iv
— and quiet, 31, i
Sorrowful, 21, ii
Speaks to the absent, 29, iii
Speaking, aversions to, 24, iii
— difficult, 27, ii ; 28, ii
Speech hasty, 22, iii ; 24, ii
Speak to him, he does not permit, 25, i
Spirits, talks of, 31, iii
Spits, 24, i ; 32, ii
Springs up from bed, 22, iv
Staring at objects, 23, v

Starts, 30, i
— frightful, sudden, 25, iv
Strikes, 24, i ; 32, ii
— all around, 32, ii
Stupefaction with delirium, 32, iii
Stupefied, 28, iv
Stupid, 29, i ; 31, ii
Stupidity, 26, ii ; 28, iv
— with delirium, 31, ii
Stupor, 26, ii
— returns, 26, iii
— muttering, 29, iv
— with delirium, 31, ii
Subject,around one only,29,ii
Suddenly, springs from bed, 22, iv
— crying out, 23, iv
— restless, 25, iii
— starts, 25, iv
Suspicious, 22, i

T.

Taciturnity, 24, iii
Taking things offered, he does not know, 22, i
— no notice, 26, ii
Talkativeness in delirium, 31, ii ; 32, ii
Talking about business, 23, i ; 30, ii
— constant, 31, ii
— to himself, 23, i
— loudly, 31, ii
— all the time, 23, i
— of animals, soldiers, etc., 23, iii
— like a drunken man, 23, iii
Talking hastily, 24, ii
— incoherently, 23, iii
— indistinctly, 23, iii
— nonsense, 23, iii
— quickly, 24, ii
— slowly, 27, ii
Talk, do not want to, 24, ii
Tall and long, he seems to be, 29, iii
Tears everything, even with his teeth, 24, i
Terrible visions, 30, i
Terrified, 33, v
Terror in expression, 21, v
Thinking, aversions to, 28, iii
— difficult, 28, ii ; 28, iii
— nothing, 27, ii
— unable to, 28, ii, iii
Thought, absorbed in the, 27, iii
— aversion to, 21, ii
— leaving, 28, iii
Thoughts, he cannot bring two together, 33, iv
— cannot be controlled, 28, iv
— difficult, 24, iv
— cannot be directed, 28, iv
— lost, 27, iii
— sitting in, 28, iv
— vanishing, 28, iii
Timid, 21, ii
Torpor, 33, iv

U.

Uncomfortable, extremely, 25, iii
Unconsciousness, 25, iv ; 26, ii

Understands no questions, 27, ii
Understood, what he says, cannot be, 23, ii
Uneasiness, 21, ii

V.

Vanishing of thoughts, 28, iii
Vexed, 22, iii
— from questions, 22, iii
Violent delirium, 32, ii
Visions, 31, v
— of beauty, 29, iv
— changing, 30, i
— terrible, 30
— of terror, 29, iv
Vivid hallucinations, 30, i
Vows fulfilling, 31, ii

W.

Wants to go from one bed into another, 21, iii
— to be dressed, 31, iv
Want of ideas, 28, i
— has none, but thirst, 20, ii ; 26, ii
Waving hands, 23, vi
Weak, intellect, 28, iii
Well, he thinks to be, 29, iv
Weep, inclined to, 21, ii ; 22, v
Whines, does not know why, 23, iv
Whistling, 31, ii
Will, has none, 20, ii
Wishes to die, 22, v
Worriment, 34
Wrong words, 27, ii

### SENSES.

**Increased sensibility**; of all the senses: *Bellad. Mur. ac.*; especially of hearing and smell: *Phosphor.*; especially to noises: *Cinchon.*; sounds, talking, odors and light are insupportable: *Nux vom.*; increased perception of the usual pains: *Bryon.*; — followed by dullness: *Rhus tox.*; perception slow, or not comprehended: *Helleb.*; dullness of all the senses: *Stramon. Sulphur*; with apathy: *Nitr. sp. d.*; as if the senses had ceased to act: *Helleb.*; with heavy eyes, and extreme weakness: *Opium*; sensorial action sunken: | *Carb. veg.*; is completely insensible as if in a dream, he neither sees nor hears: *Bellad.*; perception entirely lost: *Colchic.*; loss of senses: *Apis, Veratr.*; no complaints, has no pains when pinched:

*Phosphor.*; stupefaction: *Stramon.*, with watery eyes: *Opium*; hallucinations of sight, smell and hearing: *Stramon.*

### SENSORIUM.

**Stupefaction**; of the head with dullness of vision: *Stramon.*; —, drunkenness, and beclouding of the head: *Nux vom.*; — and dullness: *Rhus tox.*

**Confusion**; of the head: *Arnic. Rhus tox.*, cannot read nor perform the least labor: *Mercur*; — as if bound, with slow ideas: *Carb. veg.*; — and stupefaction of mind: *Secal.*; — with vertigo: *Pulsat.*

**Dullness**; difficult to think: *Stramon.*; and heavy feeling: *Gelsem. Pulsat.*; — and vacuity: | *Sulphur.*; — like a want of memory: *Pulsat.*; — and heat of head: *Camphor*; — with bruised pain in the forehead: *Pulsat.*; — with cold sweat on the forehead and hands: *Coccul.*; — and giddiness; when getting awake and sitting up, has to lie down: *Opium.*

**Weakness in the head**; if he turns it, he loses his consciousness for a moment, and after stooping, he cannot rise: *Rhus tox.*; — and stupidity: *Rhus tox.*; and lightness: *Stramon.*

**Vertigo**; like drunkenness: *Hyosc. Pulsat.*; — increasing after delirium: *Secal.*; — as if he would fall, after rising from the bed: *Rhus tox.*; — with dullness in the forehead, as if a board were before it: *Coccul.*; — with staggering and inability to stand erect; *Secal.*; — the whole day; whirling, while moving, especially the head: *Carb. veg.*; — increased by raising the head: *Arnic. Cinchon.*; — when rising up in bed: *Bryon.*; with nausea, compelling to lie down again: *Coccul.*; — as though he were swung to and fro in a cradle, or in a swing: *Ignat.*; — while lying on the back, like whirling, with qualmishness; better when lying on the side: *Mercur.*; — when standing: *Laches. Secal.*; when sitting up: *Byron. Phosph. ac. Tarax.*; falls when trying to sit up: *Phosph. ac.*; — while raising or moving the head: *Arnic.*; — on rising from lying on the back, with obscure vision: *Nux vom.*; — when moving, especially the head: *Arnic. Carb. veg. Pulsat.*; — after motions: *Bellad.*; — on rising: | *Coccul. Nux vom. Rhus tox.*; — compelling to lie down: *Bryon. Coccul.*; — in the morning on awaking, with weakness of the limbs: *Cinchon.*; —, so that he cannot rightly comprehend an idea: *Pulsat.*; — with confusion and stupidity of the head as if he would lose his senses: *Phosphor.*; — with anxiety, and glimmering before the eyes: *Bellad.*; — and headache: *Bryon.*, with heaviness: *Cinchon.*; — with obscured vision and ringing in the ears, as if there was a whirling in a circle in the brain with momentary loss of consciousness: *Nux vom.*; — with nausea and subsequent heat: *Cinchon.*; — with muscular restlessness: *Nux mosch.*; — and fainting while leaving the bed: *Opium*; — and sleepiness, as if drunk; he does not know where he is, and walks with eyes shut: *Nux mosch.*

### INTERNAL HEAD.

**Heaviness of the head**: | *Nux mosch.* ***Opium***; —, and all things seem to whirl in a circle: *Veratr.*; — with sense of empty confusion, and severe pain:

*Hyosc.*; — and dullness: *Gelsemin. Pulsat.*; — and muddleness: *Mercur.*; — like lead in the forehead, with dull pain: *Carb. veg.*; — with pressure in the brain, and desire to lie down: *Bryon.*; —, has difficulty in raising it: *Pulsat.*; — of occiput, like lead, so that the head constantly falls backwards: *Opium*; —, with intolerance of light: *Pulsat.*; — in the morning, with drunken vertigo: *Nux vom.*; — in the vertex: *Sulphur.*; — with sensation of fullness: *Sulphur.*

Humming and buzzing in the head almost the whole day: *Phosphor.*; she cannot go to sleep, because she cannot get herself together; her head feels as though scattered about, and she tosses about the bed to get the pieces together: *Baptis.*

**Headache;** dull, stupefying, with confusion of ideas: *Baptis.*; heaviness and confusion in the head: *Bellad.*; — at the base of the brain, in the forehead, and especially in the membranes of the brain: *Hyosc.*; the brain seems to be the only organ affected: *Helleb.*

**Congestion;** to the head: *Bellad. Opium. Stramon.*; during the early stage, especially in tumultuous cases, with great drowsiness, but inability to go to sleep, and frequent starting during sleep: *Bellad.*; — to the head with moderate delirium: *Apis.*; venous — with dark, red face: *Opium.*

**Throbbing;** of the carotid and temporal arteries, and also in the forehead: *Bellad.*; — in the top of head, left side; in the back of the head, or in the temples, frequently for half an hour: *Phosphor.*; — in the forehead and occiput, worse when moving: *Bryon.*; — from every movement, with heat in the head: *Laches.*; — sensation as if the head were bound with a band, with pressure: *Mercur.*

**Stupefying** pain: *Baptis.*; pressing in the forehead, and grave heaviness, with rushing sounds in the ears: *Arsen.*; heavy, stupid headache in the frontal region: *Bryon.*; — pressure in the forehead, which changes into shootings or tearings (on the left side): *Hyosc.*; pressing, contracting, stinging headache: *Bellad.*

**Pressing** outwards in the temples: *Mercur. Rhus tox.*; — the sides together; *Rhus tox.*; — in the occiput; outward — in the forehead, with pain in the supra orbital bone, mostly when touched: *Mercur.*; dull or stitching pains, worse from motion, and opening the eyes: *Bryon.*; pain in forehead and temples: *Apis. Bellad. Glonoin. Hyosc. Stramon.*

**Tearing** pain in the occiput: I *Tarax.*; on left side: *Hyosc.*; as if the brain were torn: *Rhus tox.*; in the morning on awakening, and after: *Pulsat.*

**Bruised pain,** as if compressed: *Nux vom.*; in the forehead: *Pulsat.*; in the occiput as if the cerebellum were bruised: *Rhus tox.*; pain first in the forehead, then in the occiput: *Nux mosch.*

**Headache** with the delirium: *Arsen. Colchic.*; violent pain in the forehead: *Bellad.*; — increased by moving the eyes; eyes red, prominent, staring, sparkling, brilliant, distorted or affected by spasmodic motions: *Bellad.*; — worse from opening and moving the eyes: *Bryon. Rhus tox.*; — in the forehead and eyes: *Baptis.*; does not allow opening of the eyes, followed by attacks of yawning and stretching: *Ignat.*; when waking from sleep: *Rhus tox.*; in the evening: *Bryon.*; — with lassitude: *Bryon.*

**Heat,** and burning in brain: *Calc. ostr. Phosphor*; — in head: *Camphor*, and fullness: *Sulphur*; sensation of — mostly in head: *Bryon. Rhus tox.*; — and chilliness in body: *Coccul.*; cerebral irritation predominant: *Apis. Bellad. Bryon. Cuprum. Hyosc. Laches. Opium. Stramon. Zincum*; again the 14th day: *Calc. ostr.*; inflammatory cerebral irritation; power of the senses and mental faculties impaired, speech heavy and embarrassed, they no longer recognize their own relatives, or are carried away by a furious delirium, and make attempts to escape out of their beds: *Bellad.*

### EXTERNAL HEAD.

Throbbing of arteries: *Bellad. Opium*; turns the head from side to side: *Arsen.*; frequently raises or jerks the head from the pillow: *Stramon.*; movement of occiput, grasping it with hands: *Carb. veg. Opium*; raises up the head, but it constantly falls backwards, and the mouth opens to the widest extent: *Colchic.*; inability to hold up the head; congestion (see Eyes): *Opium*; he can hardly hold the head erect, and falls asleep: *Cinchon.*

Forehead covered with cold sweat: *Colchic. Mercur. Veratr.*; head and extremities cold, body hot: *Phosphor*; great sensibility of scalp to the touch, like a boil: *Rhus tox.*; impelled to rub the forehead, with a kind of insensibility: *Veratr.*

### SIGHT AND EYES.

**Sight**; intolerance of light: *Bellad. Pulsat.*; sensibility to it: *Laches.*; shuns the light: *Mur. ac.*; hallucination of sight; all objects appear oblique or double, or smaller and further off: *Stramon.*; obscured vision with vertigo: *Nux vom.*; diminished sight and hearing, or entire loss of these senses: *Secal.*; apparent loss of sight: *Psorin. Sulphur*; blindness: | *Gelsem.* | *Stramon.* *Zincum*; loss of sight, hearing and speech: *Stramon.*

**Pupils**, much dilated, little sensitive to the light: *Carb. veg.*, and immovable: *Colchic.*; — dilated and immovable: *Stramon.*; — oftener large than small; no reaction to light; either contracted, or greatly dilated and immovable: *Bellad.*; — immovable, but sees and talks: *Stramon.*; — immovable and but slightly dilated: *Colchic.*; — dilated: *Mercur. Helleb.*, with restlessness: *Nux vom.*; — contracted, dilated or immovable, insensible to light: *Opium*; — much contracted or dilated, and insensible to light: *Cinchon.*; — first contracted and then dilated: *Pulsat.*; the left pupil contracted while the right is dilated: *Colchic.*; — contracted, or dilated, with slow respiration: *Coccul. Hyosc. Nux vom. Secal. Veratr.*; — contracted: *Arsen. Mur. ac. Stramon. Sulphur*, with cloudiness of the head: *Arnic.*

**Eyes**; bright and injected: *Glonoin. Helleb. Hyosc. Stramon.*; — glistening: *Arsen. Stramon.*, with contracted pupils: *Mur. ac.*; — brilliant, with delirium: *Lachnanth.*; — unusually bright: *Opium*; — red and sparkling, staring: *Bellad.*; — rolling about in their orbits: *Hyosc.*; — glassy: *Bellad. Opium*, and tearful: *Bryon.*; — dull: *Gelsem.*, and weak: *Mercur. Laches. Stramon.*; — without lustre: *Arsen. Hyosc. Mercur.*, and pupils without reaction to light: *Carb. veg.*; — without expression: *Bellad.*; vacant look with dilated pupils: *Helleb.*; expressionless like the — of a dying man: *Opium*; — watery: *Bryon.*, and stupid senses: *Opium*, and sunken: *Arsen.*; — heavy: *Baptis. Opium*; — dim and sleepy: *Phosph. ac.*; — prominent and turned upwards: *Arsen. Opium*; — turned awry: *Nitr. sp. d.*; — have a wild expression: *Arsen.*; hollow sunken —: *Colchic.*; — dull and sleepy: *Phosph. ac. Stramon.*; — dull and heavy: *Rhus tox.*; — turned up and lying on back: *Psorin. Sulphur*; — wild and wandering: *Secal.*; — convulsed: *Bellad.*, and prominent: *Hyosc.*, and immovable: *Opium*; — turned upwards and looking over the forehead: *Opium*; rolling of the eyes: *Bellad. Hyosc. Secal.*; austere look: *Bellad.*; — distorted: *Laches.*, and staring: *Hyosc.*; squinting: *Bellad. Hyosc. Lycop. Stramon.*

**Staring**: *Hyosc. Opium. Phosph. ac. Secal. Zincum*; —, with open eyes, at one point: *Bellad.*; — at surrounding objects: *Hyosc.*; — with watery eyes, without comprehending what occurs, or recognizing his relatives: *Opium*; — with slow comprehension, and slow answers: *Helleb.*

**Eyelids**; trembling and jerking: *Coccul.*; — open: *Bellad. Hyosc. Lycop. Opium. Stramon*, with sopor: *Lycop.*; — open, and lies speechless: *Opium*; — open, in delirium: *Arsen. Opium. Stramon. Veratr.*; — half open: *Colchic.*; — half covering the dull eyes: *Phos-*

*phor.*; — paralyzed, sunken down, either cannot, or will not open them: *Coccul. Laches. Zincum*; — heavy and full, closed as if paralyzed, drowsiness increases to coma: *Coccul.*; — drop: *Arsen. Gelsem.*; — contracted: *Bellad.*; difficult raising of the —: *Opium*; on closing the — frightened starts: *Cinchon.*, frightful phantasies: *Stramon.*, visions: *Bryon.* || *Calc. ostr.*; — injected: *Baptis. Opium*; red: *Hyosc.*; chronic soreness and inflammation of —: *Sulphur*; catarrhal ophthalmia: *Euphrasia*; — covered with pus, suppurating: *Hyosc. Zincum*; — closed with sticky matter: *Arsen.*; pale watery, swelling like a little bag over the eyes: *Kali carb.*

Eyes sunken: *Arsen. Colchic. Lycop. Phosphor.*, and heavy with blue circles: *Phosphor.*, and hollow: *Colchic. Phosphor.*; — hollow with dark circles: *Cinchon.*

### HEARING AND EARS.

**Hearing**; over sensitiveness: *Bellad. Bryon. Lycop. Mur. ac. Phosphor.*, to music: *Bryon*; every sound annoys him: *Sulphur*; noise is intolerable: *Cinchon. Coccul.*

**Ringing**: *Arsen. Coccul.*; in the head: *Arsen.*, and rushing sounds in the ear: *Opium*; buzzing, singing, rushing sounds: *Hyosc.*

**Rushing sounds**: *Hyosc. Mercur.*, like noise: *Arsen.*; like wind: *Pulsat.*, and humming: *Secale*, and thundering: *Laches.*

Illusions, hallucinations: *Stramon.*; like rain or music: *Mur. ac.*; with vertigo: *Nux vom.*

**Hardness of hearing**: *Apis. Arsen. Bryon. Phos-*

*phor.*; —, commencing with buzzing: *Hyosc.*; — in fever: *Bellad. Chlorum. Carb. veg. Hyosc. Nitr. sp. d. Phosph. ac.*

**Deafness**: *Bellad. Carb. veg. Chlorum. Hyosc. Lachnanth. Laches. Mercur. Nitr. sp. d. Phosph. ac. Psorin. Stramon. Sulphur*; — as if the ears were stopped; one or both: *Veratr.*, — with a rushing sound: *Mercur*, like the wind: *Pulsat.*; — with humming and rushing sounds in the ears: *Secal.*; — with ringing in ears and head: *Arsen.*

Great dryness in the ears, disappears rapidly after a dose of *Sulphur.* Parotid glands swollen: *Mercur.*

### SMELL AND NOSE.

**Nose.** Can endure no odors: *Sulphur*; smell and taste very acute: *Mur. ac.*; frequent bad smell: *Arsen.*: hallucinations of smell: *Stramon.*

**Dryness**; in the nose: *Bryon. Nux vom. Phosphor*; in the nostrils: *Pulsat.*; of the nose, lips and tongue: *Mur. ac.*; coming from the mouth: *Bellad.*; from the mouth and throat: *Bellad.*

Nasal mucus dried to hard crusts: *Rhus tox.*; acrid ichorous discharge excoriating the alæ nasi and upper lips, rendering them raw and sore: *Arum triph.*

**Bleeding**: *Arnic. Hyosc. Laches. Phosph. ac. Secal. Sulph. ac.*; only from right side, at night in sleep: *Veratr.*; — from the mouth and gums: *Carb. veg.*; — from the teeth and gums: *Sulphur*; — for seven days: *Sulphur*; continued —: *Nux vom.*; — frequent and copious, mostly in the evening: *Phosphor.*; hemorrhage: *Carb. veg.*; — at night: *Rhus tox.*; — in sleep: *Bryon. Mercur.*; — mostly after midnight, or in the morning: *Rhus tox.*; — mostly at three o'clock A. M., or after rising; — daily, for many days: *Bryon.*; — at the commencement on the disease: *Rhus tox.*; — with salivation: *Hyosc.* (do not give *Mercur.*); — relieves: *Rhus tox.*, does not relieve: *Phosph. ac.*

**Nostrils**; distended: *Opium*; fan-like motion of —: | *Lycop.*; — dry: *Pulsat.*, and black: *Colchic.*; — sooty, smoked: *Arsen. Chlorum. Helleb. Hyosc.*; — look smoked with dry tongue and mouth: *Chlorum.*

Nose pointed: *Veratr.*, and cold: *Camphor.*, and cold mouth: *Veratr.* (*Cina.*)

### FACE.

**Face**; cold sweat on the forehead; cool, pale, moist, thin, death-like features; at the height of disease: *Veratr.*; — cold, covered with cold sweat: *Carb. veg.*; — hot, mostly in the evening: *Bryon.*; — sweats on the right side: *Pulsat.*; — covered with sweat: *Colchic.*; — wears a happy and strange expression: *Apis*; stupid expression: *Colchic. Stramon.*; listless expression: *Rhus tox.*; besotted expression, apathic and indifferent mind: *Phosph. ac.*; stupid expression without collapse: *Helleb.*; stupid aspect, with relaxed and hanging facial muscles and lower lip: *Opium*; relaxation of the facial muscles: *Opium*, *Zincum*; trembling and jerking: *Coccul.*; convulsive trembling of lips and tongue: *Opium*; spasmodic distention: *Stramon*; distorted: *Hyosc.*; spasmodic

